# Decoupling channel count from field of view and spatial resolution in single-sensor imaging systems for fluorescence image-guided surgery

**DOI:** 10.1117/1.JBO.27.9.096006

**Published:** 2022-09-26

**Authors:** Steven Blair, Missael Garcia, Zhongmin Zhu, Zuodong Liang, Benjamin Lew, Mebin George, Borislav Kondov, Sinisa Stojanoski, Magdalena Bogdanovska Todorovska, Daniela Miladinova, Goran Kondov, Viktor Gruev

**Affiliations:** a University of Illinois at Urbana-Champaign, Department of Electrical and Computer Engineering, Urbana, Illinois, United States; b University Clinic Hospital, Ss. Cyril and Methodius University of Skopje, Department of Thoracic and Vascular Surgery, Skopje, Republic of North Macedonia; c University Clinic Hospital, Ss. Cyril and Methodius University of Skopje, Institute of Pathophysiology and Nuclear Medicine, Skopje, Republic of North Macedonia; d University Clinic Hospital, Ss. Cyril and Methodius University of Skopje, Department of Pathology, Skopje, Republic of North Macedonia; e University of Illinois at Urbana-Champaign, Beckman Institute for Advanced Science and Technology, Urbana, Illinois, United States; f University of Illinois at Urbana-Champaign, Carle Illinois College of Medicine, Urbana, Illinois, United States

**Keywords:** Image-guided cancer surgery, tumor detection, sentinel lymph node mapping, multiscale spectral imaging, stacked photodiode image sensor, pixelated optical filter

## Abstract

**Significance:**

Near-infrared fluorescence image-guided surgery is often thought of as a spectral imaging problem where the channel count is the critical parameter, but it should also be thought of as a multiscale imaging problem where the field of view and spatial resolution are similarly important.

**Aim:**

Conventional imaging systems based on division-of-focal-plane architectures suffer from a strict relationship between the channel count on one hand and the field of view and spatial resolution on the other, but bioinspired imaging systems that combine stacked photodiode image sensors and long-pass/short-pass filter arrays offer a weaker tradeoff.

**Approach:**

In this paper, we explore how the relevant changes to the image sensor and associated image processing routines affect image fidelity during image-guided surgeries for tumor removal in an animal model of breast cancer and nodal mapping in women with breast cancer.

**Results:**

We demonstrate that a transition from a conventional imaging system to a bioinspired one, along with optimization of the image processing routines, yields improvements in multiple measures of spectral and textural rendition relevant to surgical decision-making.

**Conclusions:**

These results call for a critical examination of the devices and algorithms that underpin image-guided surgery to ensure that surgeons receive high-quality guidance and patients receive high-quality outcomes as these technologies enter clinical practice.

## 1 Introduction

Near-infrared fluorescence image-guided surgery is often posed as a spectral imaging problem since surgeons must view color images of the surgical site and fluorescence images of critical anatomy. From this spectrum-centric perspective, in which cameras must capture three channels in the visible spectrum and one or more channels in the near-infrared spectrum, a large channel count is valuable because it permits the surgeon to distinguish more fluorophores and discriminate more tissue types.^1^^–^^9^ But near-infrared fluorescence image-guided surgery is rarely posed as a multiscale imaging problem even though surgeons must switch between zoomed-out images of the surgical site and zoomed-in images of the critical anatomy. From this scale-centric perspective, in which cameras must capture everything from the macroscopic to the microscopic, a camera with a large field of view and small spatial resolution is valuable because it permits the surgeon to see a larger region of the surgical site in more detail, facilitating a seamless transition from structures larger than organs to structures smaller than tissues.^5^^,^^7^

Imaging systems marketed for image-guided surgery typically collect visible and near-infrared images using a division-of-time architecture (as with the Hamamatsu PDE-NEO^10^^,^^11^ and Stryker SPY-PHI^12^) or a division-of-optical-path architecture (as with the Medtronic Elevision IR^13^^,^^14^ and Quest Spectrum^15^^,^^16^). These systems can be constructed with off-the-shelf components, conferring the notable benefit of decades of optical development, but they suffer from fundamental tradeoffs in time, space, and channel count. On the one hand, division-of-time instruments project different wavelengths onto a single sensor at different times by, e.g., switching the excitation source or emission filter, so they require additional time for each spectral channel and cannot maintain temporal coregistration between spectral channels. On the other hand, division-of-optical-path instruments split different wavelengths across multiple sensors along different optical paths with, e.g., prisms or beamsplitters, so they require additional space for each spectral channel and cannot maintain spatial coregistration between spectral channels. To permit the high frame rate and small footprint needed in both open surgery and minimally invasive surgery, imaging systems based on a division-of-focal-plane architecture have been proposed, utilizing a single sensor equipped with a filter array to sample different spectral channels at different locations, but these systems introduce the very tradeoff between the field of view, spatial resolution, and channel count that threatens the success of image-guided surgery. Consequently, these single-sensor devices typically provide three channels in the visible and one channel in the near-infrared to maximize the field of view and spatial resolution while providing the bare minimum channel count.^17^^–^^27^

What emerges within this design space is a complex relationship between the parameters that would optimize an image-guided surgery and the parameters that are practical in an imaging system. Maximum benefit to both the surgeon and patient may not be achieved via the default implementations of division-of-time, division-of-optical-path, and division-of-focal-plane instruments but may instead necessitate careful modifications to these architectures. An example comes from a hexachromatic bioinspired imaging system for image-guided cancer surgery that was recently presented by Blair et al.^28^ This imaging system captures three channels in the visible spectrum and three channels in the near-infrared spectrum, facilitating the detection of multiple fluorophores in the operating room, but it captures those six channels, not with the six pixels required by a conventional image sensor but with only two pixels provided by a stacked photodiode image sensor. This device thus captures more spectral channels in fewer pixels—maintaining the balance between the channel count demanded from the spectrum-centric perspective and the field of view and spatial resolution demanded from the scale-centric perspective.

In this paper, we explore how the tradeoff between spectral performance and multiscale performance is affected by the design choices for division-of-focal-plane imaging systems by comparing the six-channel imaging system described in Ref. 28 with the four-channel imaging systems that have long been favored by researchers and manufacturers alike.^17^^–^^27^ We do so by fixing the largest scale available to the camera (i.e., fixing the field of view) and evaluating performance at the smallest scale available to the camera (i.e., evaluating performance at the spatial resolution). A discussion of the two imaging systems is provided in Sec. 2, a discussion of the associated image processing routines is provided in Sec. 3, and a framework for comparing the two systems is presented in Sec. 4. Methods and results are then provided in Secs. 5–6.

## 2 Imaging System

A surgeon performing a complex operation should have instantaneous feedback, so the video feed guiding their incisions should offer both a low latency and a high frame rate. The imaging system must thus capture, in a single shot, all relevant information across all relevant scales since there is no time to collect and fuse different images at different scales (as proposed in Ref. 29). If the surgeon zooms out, they will visualize the entire surgical site but will not perceive the most detail in it, yet if the surgeon zooms in, they will perceive the most detail in the surgical site but will not visualize the entirety of it.^30^ The images collected at these extreme levels of magnification, and any images taken at the intermediate levels in between, illustrate a tradeoff between the field of view (How much of the surgical site can the surgeon visualize?) and the spatial resolution (How much detail in the surgical site can the surgeon perceive?). This tradeoff may be acceptable in some cases; the surgeon may, for example, want to determine the location of one or more small lesions at the macroscopic scale and then determine the extent of those lesions at the microscopic scale.^31^ The former task can utilize a larger field of view at the expense of a degraded spatial resolution, whereas the latter task can utilize a smaller spatial resolution at the expense of a degraded field of view. But this tradeoff may be unacceptable in other situations; the surgeon may instead want to monitor the boundary of their resection against the path of a nearby nerve, utilizing a more aggressive margin where tissue preservation is not necessary and a less aggressive margin where it is.^32^^–^^34^ A larger field of view may be required to frame both the lesion and the nerve, but a smaller spatial resolution may be required to trace the diffuse boundary of the lesion and the outstretched branches of the nerve. Flexibility is also necessitated in applications like near-infrared spectroscopy where it may be critical to examine vital quantities, related perhaps to patient hemodynamics, across particular regions^35^ or above especially sensitive structures.^36^^,^^37^

**Fig. 1:**
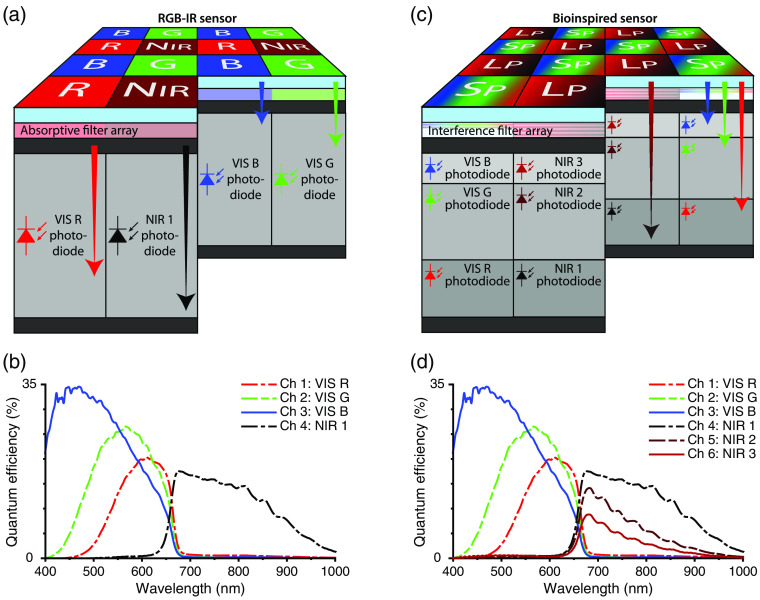
The structure and function of an RGB-IR camera and a bioinspired camera. (a) A red/green/blue/near-infrared camera (or RGB-IR camera) is constructed from a single layer of silicon photodiodes topped with an additional layer of four different absorptive filters. The silicon photodiodes exhibit a broadband response spanning the visible (VIS) and near-infrared (NIR), while the absorptive filters exhibit a relatively narrow transmission tuned to blue light, green light, red light, or NIR light. Since each photodiode absorbs all colors of light while each absorptive filter passes a different color of light, the camera can provide, in every 2×2  pixel neighborhood, three observations in the VIS spectrum and one observation in the NIR spectrum. (b) The quantum efficiencies presumed for the RGB-IR camera that was used in this study, illustrating the tetrachromatic (“four-color”) vision offered. The curves have been adopted from Ref. 28, consistent with the methods in Ref. 38. (c) A bioinspired long-pass/short-pass camera (or bioinspired camera) makes two substitutions compared with an RGB-IR camera: First, it replaces the single layer of silicon photodiodes with three layers of such photodiodes (each sensitive to shorter wavelength, longer wavelengths, or intermediate wavelengths), and second, it replaces the layer of four different absorptive filters with a layer of two different interferences filters (each serving as a short-pass filter tuned for VIS light or a long-pass filter tuned for NIR light). Since each photodiode absorbs and each interference filter passes a different color of light, the camera can provide, in every 2×1  pixel neighborhood, three observations in the VIS spectrum and three observations in the NIR spectrum. (d) The quantum efficiencies evaluated for the bioinspired camera that was used in this study, illustrating the hexachromatic (“six-color”) vision offered. The curves have been adopted from Ref. 28.

The tradeoff between field of view and spatial resolution is exacerbated by the conventional architecture of single-sensor cameras. The red/green/blue/near-infrared architecture, also known as the RGB-IR or RGB-NIR architecture, captures three visible channels that facilitate color imaging of the surgical site and one near-infrared channel that facilitates fluorescence imaging of a single surgical target. Fabrication entails the formation of silicon photodiodes on a rectangular grid, providing sensitivity from 400 to 1100 nm, followed by deposition of optical filters in a 2×2  pixel pattern, permitting discrimination between blue light, green light, red light, and near-infrared light [Figs. 1(a) and 1(b)]. This design adds tension to the existing tradeoff between the field of view and the spatial resolution; each channel is sampled by a quarter of the pixel array, so each channel’s spatial resolution is limited to a fraction of the pixel array’s spatial resolution—requiring a further restriction to the field of view to compensate for the spatial resolution. But it also adds a new dimension to the balancing act; the addition of multiple near-infrared channels requires the addition of multiple near-infrared pixels, meaning that the detection of additional surgical targets entails an additional tradeoff with the field of view and the spatial resolution.

The tradeoff between field of view, spatial resolution, and channel count arises because the sensor makes observations over a finite area and at a finite spatial sampling rate, so an improvement in these three quantities can be achieved by modulating either the area or the spatial sampling rate. One option is to increase the area, sizing up the sensor while fixing the pixel’s size. As the sensor becomes larger, manufacturing tolerances become looser, decreasing the spatial uniformity, and defects become likelier, increasing the failure rate. This may entail additional calibration of the sensor in the best case and outright replacement in the worst, increasing cost and decreasing reliability.^39^ An increase in sensor size also demands an increase in camera size, which can make some instruments, like cart-based systems for open surgeries, less convenient and can make other instruments, like endoscopic systems for minimally invasive surgery, impossible. Another option is to increase the spatial sampling rate, sizing down the pixels while fixing the sensor’s size. As the pixels become smaller, their dynamic range is reduced on both the low end and the high end; a smaller number of photons passes through each photodiode due to the reduced cross-sectional area, degrading sensitivity to small signals, and a smaller number of photoelectrons can accumulate on each photodiode due to the reduced junction capacitance, degrading sensitivity to large signals.^39^ Additionally, their noise performance is harmed both spatially and temporally; since smaller pixels require smaller features, it becomes difficult to implement the isolation trenches that reduce crosstalk between pixels,^40^ causing photoelectrons to stochastically migrate between pixels, and the pinned photodiodes that reduce image lag between frames,^41^ causing photoelectrons to stochastically migrate between frames.

A solution to the original tradeoff between field of view and spatial resolution and the additional tradeoff with channel count is posed by an alternative architecture for single-sensor cameras. The bioinspired long-pass/short-pass architecture captures three visible channels that facilitate color imaging of the surgical site and three near-infrared channels that facilitate fluorescence imaging of three surgical targets. There are two key differences between the RGB-IR sensor and the bioinspired sensor. First, an RGB-IR sensor utilizes a single layer of silicon photodiodes with sensitivity across the visible and near-infrared, whereas the bioinspired sensor utilizes three layers of silicon photodiodes. Longer wavelengths penetrate farther than shorter wavelengths upon transmission through silicon, so the bottommost photodiodes are more sensitive to longer wavelengths and the topmost photodiodes are more sensitive to shorter wavelengths [Fig. 1(c)]. Second, an RGB-IR sensor utilizes four optical filters that discriminate blue, green, red, and near-infrared, whereas the bioinspired sensor utilizes two optical filters. A short-pass filter passes visible light below 700 nm and rejects longer wavelengths, whereas a long-pass filter passes near-infrared light above 700 nm and rejects shorter wavelengths [Fig. 1(c)]. As a result of these design choices, the three photodiodes under the short-pass filters detect the three colors of visible light, whereas the three photodiodes under the long-pass filters detect three “colors” of near-infrared light [Figs. 1(c) and 1(d)]. This architecture does not obviate the tradeoff between field of view and spatial resolution, but it does weaken the tradeoff. An RGB-IR sensor makes one observation of each channel in each 2×2  pixel block, whereas the bioinspired sensor makes two observations of each channel in each 2×2  pixel block. Likewise, it does not eliminate the impact of additional spectral channels on the field of view and spatial resolution, but it does lessen the impact. An RGB-IR sensor suffers in field of view and spatial resolution for every single channel added, whereas the bioinspired sensor suffers in field of view and spatial resolution for every three channels added.

## 3 Demosaicing Routine

**Fig. 2:**
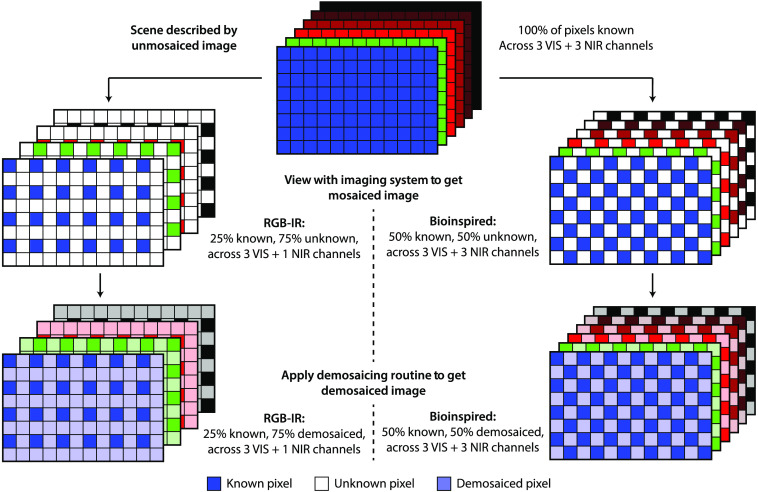
The need for demosaicing routines. While blue light, green light, red light, and near-infrared light are reflected from every point in a scene (as illustrated in the unmosaiced image in the top row with all pixels known), these colors of light are not observed at every pixel in a single-sensor imaging system (as illustrated in the mosaiced images in the middle row with some pixels known and others unknown). As a result, demosaicing routines must generate demosaiced images (like those in the bottom row) that can be displayed to an end-user or consumed by a computer. Yet even though the loss of spatial information during the imaging process can be mitigated by the demosaicing routine, the loss of spectral information cannot. Consequently, an RGB-IR camera will only be able to provide three VIS channels and one NIR channel, even after the demosaicing routine has been applied, and a bioinspired camera will still be able to provide three VIS channels and three NIR channels.

Since a surgeon who has turned their eyes toward the surgical field will perceive the color of light at every point on the patient, that surgeon, when instead directing their attention toward a surgical display, will expect to see the color of light at every point in the video feed (Fig. 2, top row). However, single-sensor cameras are rarely able to observe every color at every point as has been noted with the RGB-IR sensor, which captures each channel at just one-quarter of its pixels, and the bioinspired sensor, which captures each channel at only one-half of its pixels (Fig. 2, middle row). This necessitates a demosaicing routine that can estimate the unmosaiced image that would be perceived by the surgeon with a demosaiced image that can actually be shown to them upon analysis of the mosaiced image captured by the imaging system (Fig. 2, bottom row).

The need for a demosaicing routine that can recover the intensity at some pixels because the imaging system cannot sense the intensity at those pixels implies a loss in spatial resolution, but if a demosaicing routine was discovered that could infer, without error, the pixels where a channel is unknown from the pixels where a channel is known, then it would be impossible to distinguish between an unmosaiced image captured at a high spatial resolution and a demosaiced image captured at a low spatial resolution—suggesting that the demosaicing routine recovered the spatial resolution. Of course, all demosaicing routines rely on assumptions about the scene, assumptions that cannot possibly hold for every scene, and will thus be characterized by some nonzero error. However, a demosaicing routine can be designed, with prior knowledge, to make the appropriate assumptions about the scene—permitting that demosaicing routine to recover structure that is observed only in part. In this way, demosaicing routines cannot improve spatial resolution in a deterministic sense—enhancing the rate at which observations are made without any error—but can improve spatial resolution in a stochastic sense—enhancing the rate at which observations are made with bounded error. As a result, the demosaicing routine can weaken the tradeoff between spatial resolution, field of view, and channel count by permitting a lower spatial resolution to be accepted during the imager design and a higher spatial resolution to be recovered during the image processing.

Demosaicing routines have received considerable interest in the context of conventional imaging where it is possible to exploit statistical models that describe, e.g., how likely a color is to occur or how the amount of red and blue light correlates with the amount of green light.^42^^,^^43^ But they have received little interest in the context of biomedical imaging where it is not clear that such statistical models are appropriate or accurate. First, a surgeon is often just as interested in a rare-looking structure as a common-looking one since the rare-looking structure may represent a pathological condition. As a result, a bias away from rare colors toward common ones could cause the surgeon to miss a rare-looking structure that is relevant to the operation. Second, the visible channels and the near-infrared channels will be uncorrelated by design since the visible channels represent what the surgeon would normally perceive and the near-infrared channels represent what the surgeon would not. As a result, a bias toward correlated channels will corrupt the information in each channel. Toward this end, the conservative assumption should be made that the channels are statistically independent.

A family of demosaicing routines that is compatible with dual-mode visible/near-infrared imaging systems like the RGB-IR sensor and the bioinspired sensor but does not make assumptions on the correlation between the visible channels and the near-infrared channels has already been proposed in Ref. 38. These demosaicing routines recognize that each channel is fully defined by a complicated function of two variables, the row number and column number, that can be locally approximated by much simpler interpolating polynomials taken in one of several variations. By choosing between polynomials or splines defined in one or two dimensions and of the first or third degree, the designer of an imaging system can select a demosaicing routine that best matches the scenes of interest. For example, polynomials, which can be fitted to small portions of a row, column, or image without undue assumptions on the image’s derivatives, suit those surgical sites where the transition between tissues is abrupt or erratic, whereas splines, which are usually fitted to the entire span of a row, column, or image under restrictive assumptions on the image’s derivatives, suit the remaining surgical sites where tissues transition continuously and smoothly. Likewise, polynomials and splines in one dimension may not recognize features with an indefinite orientation that those in two dimensions would, like a blood vessel perfused with indocyanine green that snakes in every direction through the surgical site, and polynomials and splines of the first degree may not reconstruct features with rapidly-varying intensities that those of third degree would, like a nerve labeled with Oxazine 4 that branches densely within the surgical site. However, polynomials and splines in one dimension or of the first degree can be fitted with far less data than those in two dimensions or of the third degree, making them more robust to noise.

## 4 Comparing the Imaging Systems and Demosaicing Routines

An imaging system operates at its spatial resolution when the imaging system can just distinguish two point sources. The imaging system, under such conditions, will see two intensity maxima produced by the point sources and one intensity minimum between them—three intensity extrema in total. The spatial resolution may be fundamentally limited by the aperture stop of the imaging lens, which determines the diffraction-limited spot size, but it may also be limited by the image sensor and demosaicing routine.

The image sensor observes the intensity at a subset of known pixels, so the spatial resolution can be upper bounded by the smallest neighborhood of known pixels that will permit discrimination of two point sources. For example, an image sensor without a filter array, where the pixels belong to a single channel, can resolve two point sources in the span of three pixels since the image sensor can identify the three intensity extrema associated with the point sources from the three observations taken at adjacent pixels. However, an image sensor with a filter array, where the pixels belong to multiple channels, may require additional pixels to resolve the point sources since three pixels from any given channel may be interspersed with one or more pixels from a different channel. In turn, the demosaicing routine predicts the intensity at the remaining subset of unknown pixels, so the spatial resolution can be enhanced if a smaller neighborhood of both known and unknown pixels can still permit discrimination of two point sources. For example, a demosaicing routine that was capable of divining the value of every unknown pixel from the values at the known pixels could identify the three intensity extrema associated with two point sources even if they occurred at unknown pixels. However, a demosaicing routine that was forced to guess, at random, could only identify the three intensity extrema associated with the point sources if they occurred at known pixels.

It is worth noting, though, that the spatial resolution of the image sensor depends on the orientation of the point sources since the observations of the known pixels may not be equally spaced along rows, columns, and diagonals and that the spatial resolution enhancement from the demosaicing routine depends on the context of the point sources since the predictions of the unknown pixels may not be equally valid in all situations. What’s more, the spatial resolution will further vary from channel to channel because the wavelength-dependent optical properties associated with the tissue^44^^,^^45^ will induce a wavelength-dependent pattern in the light intensity^46^^–^^48^ as different wavelengths penetrate different distances—meaning that signals may be generated by, but must also be visualized from, sources at up to several millimeters depth when working in the near-infrared spectrum.^49^ This suggests that the spatial resolution of the imaging system should not be judged from images of point sources in a contrived configuration but should instead be judged from images of the real world, in all its complexity. This suggests, in consequence, that the spatial resolution cannot be defined in the traditional sense since image-guided surgery offers no analog to the point source.

Fortunately, the spatial resolution can be evaluated in a different way. Consider an image captured by a division-of-focal plane image sensor and processed by a demosaicing routine: if the image sensor and demosaicing routine can reproduce small features, then any unmosaiced images and demosaiced images should exhibit small deviations—indicating that a proxy for the spatial resolution is provided by the error between the two images. In this way, a comparison of two imaging systems or demosaicing routines can be facilitated by a comparison of the actual deviation between the unmosaiced image that describes a scene and the demosaiced images that are ultimately observed. This comparison can be done with the sum of absolute errors, taken over all pixels, which accounts for both the vanishing error from known pixels captured by the imaging system and the nonvanishing error from unknown pixels filled in by the demosaicing routine, or it can be done with the mean of absolute errors, taken over unknown pixels, which downplays the role performed by the imaging system in favor of that performed by the demosaicing routine.^38^ Since the variation in these two measures tracks the variation between the unmosaiced image and the demosaiced image in a simple and proportional manner, they provide some transparency into how a computer program developing a surgical strategy will be affected by the combined effect of the imaging system and demosaicing routine. Alternatively, a comparison of two imaging systems or demosaicing routines can be facilitated by a comparison of the perceived deviation between the unmosaiced image and the demosaiced images. This can be done with the 99th percentile of the structural dissimilarity index, taken over unknown pixels, which describes the change in texture in the vicinity of those pixels filled in by the demosaicing routine, or the 99th percentile of the color difference, also taken over unknown pixels, which describes the change in color at those pixels.^38^ Since the variation in these two measures tracks the variation between the unmosaiced image and the demosaiced image in a complex but perceptually consistent manner, they provide a window into how a human observer thinking through their own surgical approach will be affected as an image is captured and processed.

While the proposed measures of actual deviation are well defined for both the visible channels and the near-infrared channels, the proposed measures of perceived deviation do require some special consideration. Since the RGB-IR camera captures a single near-infrared channel, it returns images in a monochromatic format that precludes display in full color, while the bioinspired camera, which instead captures a triplet of near-infrared channels, returns images in a trichromatic format that permits such a display.^28^ That being said, the 99th percentile of the structural dissimilarity can be pooled across the visible channels or the near-infrared channels so that it can be defined regardless of the channel count, whereas the 99th percentile of the color difference must be computed over three visible channels or three near-infrared channels so that it is only defined for a trichromatic channel configuration.^38^ The structural dissimilarity can thus be presented for both the visible channels and the near-infrared channels from both the RGB-IR sensor and the bioinspired sensor, but the color difference must only be presented for the visible channels from the RGB-IR sensor in addition to the visible channels and near-infrared channels from the bioinspired sensor. In this way, the structural dissimilarity for the near-infrared channels describes the change in texture perceived in those channels by a human operator that is viewing them in either a monochromatic format or a trichromatic format, whereas the color difference for the near-infrared channels describes the change in color perceived in those channels by a human operator viewing them in a trichromatic format. Note that the 99th percentile acts as an upper bound on these metrics, ensuring that the rare pixels associated with disease do not perform worse on them.

## 5 Preclinical Dataset

To explore the performance of the described image sensors and demosaicing routines on a scene that approaches that of image-guided surgery, images from a preclinical model of breast cancer were collected and analyzed. This study followed protocols approved by the University of Illinois Institutional Animal Care and Use Committee.

### 5.1 Data Collection

A female mouse (J:NU, The Jackson Laboratory; 2 to 3 months, 20 to 25 g) with a breast tumor (4T1, American Type Culture Collection; delivered via subcutaneous injection and grown to 1-cm diameter) was used in the study and was imaged before, during, and after tumor resection. IRDye 800CW Maleimide (100  μL at 11.91  μg per mL phosphate-buffered saline) was administered via injection into the retro-orbital sinus and allowed to accumulate in the tumor over a 24-h period. The mice were anesthetized via isoflurane inhalation; the tumors were imaged through the skin; the mice were sacrificed via cervical dislocation; and the tumors were imaged through an incision.

**Fig. 3:**
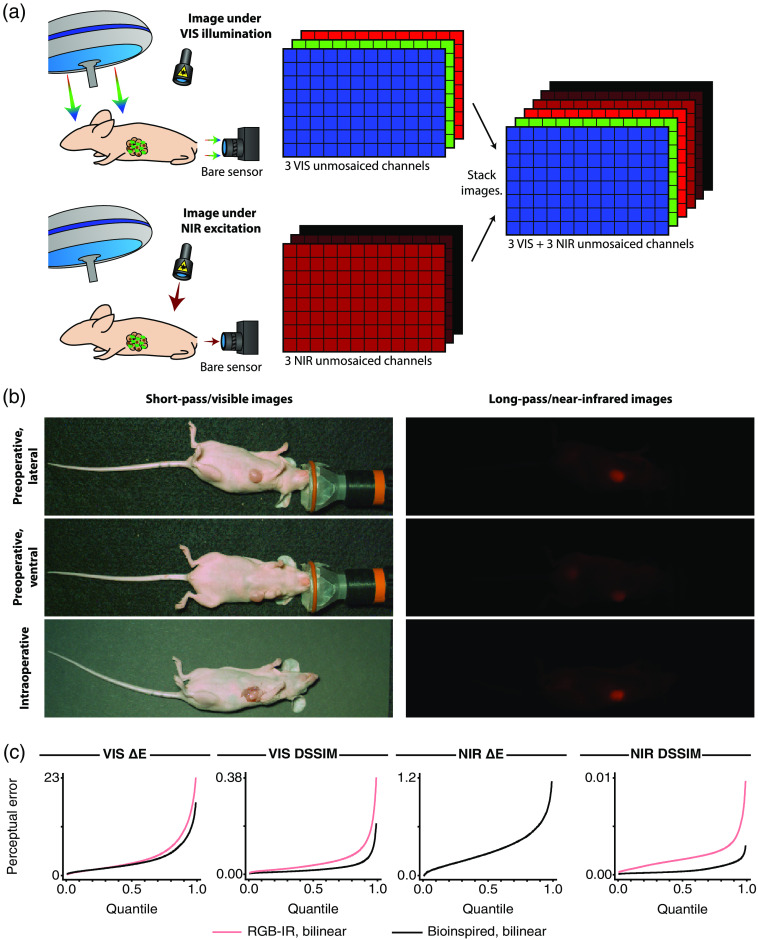
The experimental setup and unmosaiced images for the preclinical dataset along with quantile functions for the perceptual metrics. (a) A mouse with a breast tumor was administered IRDye 800CW Maleimide so that the breast tumor could be identified with near-infrared fluorescence. Raw images were collected using a stacked photodiode image sensor equipped with no filter array, with illumination provided by either a VIS source alone or an NIR source alone. Unmosaiced images were then constructed by stacking the images taken under white light illumination alone and the images taken under near-infrared illumination alone. (In this illustration, the light sources and the image sensor have been positioned for conceptual clarity; during the actual experiment, though, the light sources and the image sensor were all positioned above the mouse.) (b) Three unmosaiced images were included in the dataset: two images showing the tumor before surgery through the skin and one image showing the tumor during surgery through an incision. Each image consisted of one frame and 399×144  pixels. (c) Quantile plots for visible color difference (VIS ΔE), visible dissimilarity index (VIS DSSIM), near-infrared color difference (NIR ΔE), and near-infrared dissimilarity index (NIR DSSIM) were generated for an RGB-IR imaging system with bilinear demosaicing and a bioinspired imaging system with bilinear demosaicing. Each point indicates the proportion of pixels that have been assigned a value for a metric that is less than or equal to a given value for that metric. NIR ΔE could not be computed for the RGB-IR imaging system since only one of three total near-infrared channels could be captured by that camera.

A custom camera^28^ including a stacked photodiode image sensor with no filter array was used to capture images; the camera was equipped with an emission filter (NF03-785E, Semrock). The mouse was first illuminated by a white light source (LED-6500T, Genaray, with Cool Mirror Film 330, 3M), and an image was captured—providing a three-channel VIS image [Fig. 3(a)]. The mouse was then illuminated by a near-infrared source (I0785MU6000M4S, Innovative Photonic Solutions), and another image was captured—providing a three-channel NIR image [Fig. 3(a)]. The spectral power distribution of the white light source was restricted to wavelengths below 700 nm, so the images collected under white light illumination could be treated like images collected from a stacked photodiode image sensor covered entirely with short-pass filters. Likewise, the spectral power distribution of the near-infrared source was restricted to wavelengths above 700 nm and the spectral power distribution of the near-infrared fluorophore experienced a Stokes shift to even longer wavelengths, so the images collected under near-infrared illumination could be treated like images collected from a stacked photodiode image sensor covered entirely with long-pass filters. By stacking the three-channel white-light images and the three-channel near-infrared images and applying a pre-processing routine to the result, a full-resolution unmosaiced image could thus be formed with three short-pass observations and three long-pass observations at every pixel [Fig. 3(a), Supplementary Material].

This process yielded three unmosaiced images that were included in the dataset [Fig. 3(b)]: a pair of preoperative images of the mouse’s lateral aspect and ventral aspect, portraying the tumor through unbroken skin, and a single intraoperative image, portraying the tumor through an incision into the skin. Each image consisted of one frame and 399×144  pixels derived from a video consisting of 100 frames and 448×674  pixels; the frame was taken as the first frame of the video since the remaining frames contained no additional action, and the region-of-interest was taken around the mouse since the remaining pixels contained no additional content. This ensured that the analysis was restricted to, and the conclusions reflect, the portion of the scene that is most relevant to image-guided surgery.

Two mosaiced images were produced after each unmosaiced image in the dataset was properly sampled: (1) the mosaiced image that a bioinspired camera would see, with three short-pass observations and three long-pass observations covering different halves of the array, and (2) the mosaiced image than an RGB-IR camera would see with blue observations, green observations, red observations, and near-infrared observations covering different quarters of the array. A set of demosaiced images were then computed by applying demosaicing routines based on (a) one-dimensional linear polynomials/splines, (b) one-dimensional cubic polynomials, (c) one-dimensional cubic splines, (d) two-dimensional cubic polynomials, and (e) two-dimensional cubic splines. A comparison of the unmosaiced image and these demosaiced images at a subset of pixels identified as the foreground finally enabled an evaluation of the imaging systems and demosaicing routines (Supplementary Material).

### 5.2 Data Analysis

Tables 1 and 2 present the sum of absolute errors, mean absolute errors, structural dissimilarities, and color differences when examining the preclinical dataset across image sensors and demosaicing routines. By comparing the columns for the bioinspired sensor with those for the RGB-IR sensor, it can be seen that the bioinspired sensor outperforms the RGB-IR sensor in every metric; this conclusion is consistent with the results from Ref. 38.

**Table 1:** Sum of absolute error (SAE) for preclinical dataset. Statistics were pooled from three videos containing one frame each. Unmosaiced channels were constructed by illuminating the subject with either white light or fluorescence excitation and capturing a frame using a stacked photodiode image sensor with no filter array.

Metric	Bioinspired sensor					RGB-IR sensor				
	1D bilinear	1D bicubic poly	1D bicubic spline	2D bicubic poly	2D bicubic spline	1D bilinear	1D bicubic poly	1D bicubic spline	2D bicubic poly	2D bicubic spline
SAE (VIS R)	$6.18\times 10^{2}$	$6.06\times 10^{2}$	$6.23\times 10^{2}$	$5.90\times 10^{2}$	$6.45\times 10^{2}$	$10.99\times 10^{2}$	$10.99\times 10^{2}$	$11.56\times 10^{2}$	$11.77\times 10^{2}$	$11.56\times 10^{2}$
SAE (VIS G)	$4.27\times 10^{2}$	$4.05\times 10^{2}$	$4.11\times 10^{2}$	$3.80\times 10^{2}$	$4.13\times 10^{2}$	$7.86\times 10^{2}$	$7.61\times 10^{2}$	$7.91\times 10^{2}$	$8.49\times 10^{2}$	$7.91\times 10^{2}$
SAE (VIS B)	$5.39\times 10^{2}$	$5.26\times 10^{2}$	$5.33\times 10^{2}$	$5.09\times 10^{2}$	$5.54\times 10^{2}$	$9.71\times 10^{2}$	$9.55\times 10^{2}$	$9.83\times 10^{2}$	$10.12\times 10^{2}$	$9.83\times 10^{2}$
SAE (NIR 1)	$5.30\times 10^{1}$	$5.12\times 10^{1}$	$5.18\times 10^{1}$	$5.12\times 10^{1}$	$5.49\times 10^{1}$	$9.13\times 10^{1}$	$8.79\times 10^{1}$	$9.02\times 10^{1}$	$9.16\times 10^{1}$	$9.02\times 10^{1}$
SAE (NIR 2)	$2.95\times 10^{1}$	$3.01\times 10^{1}$	$3.07\times 10^{1}$	$3.08\times 10^{1}$	$3.32\times 10^{1}$	—	—	—	—	—
SAE (NIR 3)	$2.41\times 10^{1}$	$2.50\times 10^{1}$	$2.57\times 10^{1}$	$2.58\times 10^{1}$	$2.78\times 10^{1}$	—	—	—	—	—

**Table 2:** Mean absolute error (MAE), 99th percentile structural dissimilarity index (DSSIM), and 99th percentile color difference (ΔE) for preclinical dataset. The bioinspired sensor could be evaluated under the full set of metrics since it could capture three visible channels and three near-infrared channels, but the RGB-IR sensor could only be evaluated under a subset of metrics since it could capture just three visible channels and one near-infrared channel. A dash (“—”) indicates those cases where a statistic could not be computed since a camera did not capture all relevant channels.

Metric	Bioinspired sensor					RGB-IR sensor				
	1D bilinear	1D bicubic poly	1D bicubic spline	2D bicubic poly	2D bicubic spline	1D bilinear	1D bicubic poly	1D bicubic spline	2D bicubic poly	2D bicubic spline
MAE (VIS R)	$2.43\times 10^{-2}$	$2.38\times 10^{-2}$	$2.45\times 10^{-2}$	$2.31\times 10^{-2}$	$2.53\times 10^{-2}$	$2.88\times 10^{-2}$	$2.88\times 10^{-2}$	$3.02\times 10^{-2}$	$3.08\times 10^{-2}$	$3.02\times 10^{-2}$
MAE (VIS G)	$1.68\times 10^{-2}$	$1.59\times 10^{-2}$	$1.61\times 10^{-2}$	$1.49\times 10^{-2}$	$1.62\times 10^{-2}$	$2.06\times 10^{-2}$	$1.99\times 10^{-2}$	$2.07\times 10^{-2}$	$2.22\times 10^{-2}$	$2.07\times 10^{-2}$
MAE (VIS B)	$2.11\times 10^{-2}$	$2.06\times 10^{-2}$	$2.09\times 10^{-2}$	$2.00\times 10^{-2}$	$2.17\times 10^{-2}$	$2.54\times 10^{-2}$	$2.50\times 10^{-2}$	$2.57\times 10^{-2}$	$2.65\times 10^{-2}$	$2.57\times 10^{-2}$
MAE (NIR 1)	$20.85\times 10^{-4}$	$20.12\times 10^{-4}$	$20.34\times 10^{-4}$	$20.12\times 10^{-4}$	$21.58\times 10^{-4}$	$23.90\times 10^{-4}$	$23.01\times 10^{-4}$	$23.60\times 10^{-4}$	$23.96\times 10^{-4}$	$23.60\times 10^{-4}$
MAE (NIR 2)	$11.60\times 10^{-4}$	$11.83\times 10^{-4}$	$12.08\times 10^{-4}$	$12.09\times 10^{-4}$	$13.04\times 10^{-4}$	—	—	—	—	—
MAE (NIR 3)	$9.48\times 10^{-4}$	$9.82\times 10^{-4}$	$10.10\times 10^{-4}$	$10.13\times 10^{-4}$	$10.92\times 10^{-4}$	—	—	—	—	—
DSSIM (VIS)	0.1980	0.1969	0.2020	0.1948	0.2246	0.3791	0.3849	0.4025	0.3794	0.4025
ΔE (VIS)	17.0463	17.1692	17.3256	17.3665	18.3730	22.9444	22.6101	22.9091	22.5750	22.9091
DSSIM (NIR)	0.0029	0.0025	0.0026	0.0026	0.0030	0.0096	0.0078	0.0080	0.0101	0.0080
ΔE (NIR)	1.1474	1.1252	1.1423	1.1425	1.2163	—	—	—	—	—

To observe how the demosaicing routine influenced the actual error once a given sensor was selected, a rank was assigned to each demosaicing routine according to its performance on the sum of absolute errors or the mean absolute error, with the sum or the mean computed over the pooled visible channels or the pooled near-infrared channels. For both the bioinspired sensor and the RGB-IR sensor, the sum of absolute error and the mean absolute errors were minimized with bicubic polynomial interpolation; however, the bioinspired sensor yielded better performance from a two-dimensional routine for the visible channels and a one-dimensional routine for the near-infrared channels, whereas the RGB-IR sensor yielded better performance from a one-dimensional routine regardless of the channels of interest.

To further explore how this design choice affected the perceived error, an individual rank was assigned to each demosaicing routine according to its performance on each perceptual metric, and an average rank was computed for each demosaicing routine over all perceptual metrics. The numbers from this process, when compared across demosaicing routines for an individual sensor, facilitate the ranking of the demosaicing routines assuming, first, that visible and near-infrared spectra are equally important and, second, that color and texture reproduction are also equally important. For both sensors, it was found that a two-dimensional bicubic polynomial demosaicing routine performed the second best among all routines whereas a one-dimensional bicubic polynomial demosaicing routine performed the best.

Figure 3(c) presents seven quantile functions corresponding to the four perceptual error metrics (visible color difference, visible structural dissimilarity, near-infrared color difference, and near-infrared structural dissimilarity) evaluated for the two sensors and their associated bilinear demosaicing routines. Each point on these curves indicates the proportion of pixels (the “quantile” indicated along the x-axis) that have been assigned a value for a metric that is less than or equal to a given value for that metric (the “perceptual error” indicated along the y-axis); therefore, a vertical drop in the curve indicates, not just a new bound on a single pixel, but also a new bound on an entire subpopulation of pixels.

For the visible color difference, the quantile function for the bioinspired sensor lies above the quantile function for the RGB-IR sensor for the ∼20% of pixels with the smallest color difference but falls below the quantile function for the RGB-IR sensor for the remaining ∼80% of pixels with a larger color difference. However, the transition between these regimes lies near a color difference of 1.32 to 1.33, which narrowly exceeds the just noticeable difference of 1.00, indicating that pixels where the bioinspired sensor underperforms the RGB-IR sensor will be largely undetectable to a human observer. For the visible structural dissimilarity index and the near-infrared structural dissimilarity index, the story is simpler, with the quantile functions for the bioinspired sensor lying wholly below the quantile functions for the RGB-IR sensor. As a result, the transition to a bioinspired sensor from an RGB-IR sensor offers a maximum improvement of 28% for the visible color difference, 54% for the visible structural dissimilarity index, and 80% for the near-infrared structural dissimilarity index, with any impairment remaining nearly imperceptible.

It is interesting to note that the gap between the quantile function for the bioinspired sensor and the quantile function for the RGB-IR sensor generally increases as the quantile increases, indicating that the image undergoes the largest improvement in quality at those pixels that exhibit the largest amount of error. A large error during demosaicing is typically associated with a boundary between unlike pixels, which may further correspond to a boundary between diseased tissue and healthy tissue. As a result, a substantial decrease in the maximal error may permit better discrimination between the most clinically relevant tissue types.

## 6 Clinical Dataset

To explore the performance of the image sensors and demosaicing routines on a scene that accurately reflects that of image-guided surgery, images from clinical cases of breast cancer were collected and analyzed. This study followed protocols approved by the Institutional Review Board at the University of Illinois at Urbana-Champaign and the Agency for Drugs and Medical Instruments in Skopje, Republic of North Macedonia, and it included only those patients who provided informed consent.

### 6.1 Data Collection

Seven women [58±12  years (mean ± standard deviation)] with breast tumors (diagnosed as early or progressive) were recruited into the study and were imaged during sentinel lymph node mapping. Technetium-99m-labeled human serum albumin colloid (Tc99m-HSA colloid; 834  μCi), indocyanine green (ICG; 2 mL at 0.5 mg ICG per mL saline), and methylene blue (MB; 1 mL at 10 mg MB per mL water) were administered via injection into the tumor area and allowed to accumulate in the lymph nodes over a 10- to 15-min period. The patient was placed under general anesthesia; an incision was made into the tumor area; sentinel lymph nodes were identified via radioactivity and visible contrast; and sentinel lymph nodes were imaged via near-infrared fluorescence.

**Fig. 4:**
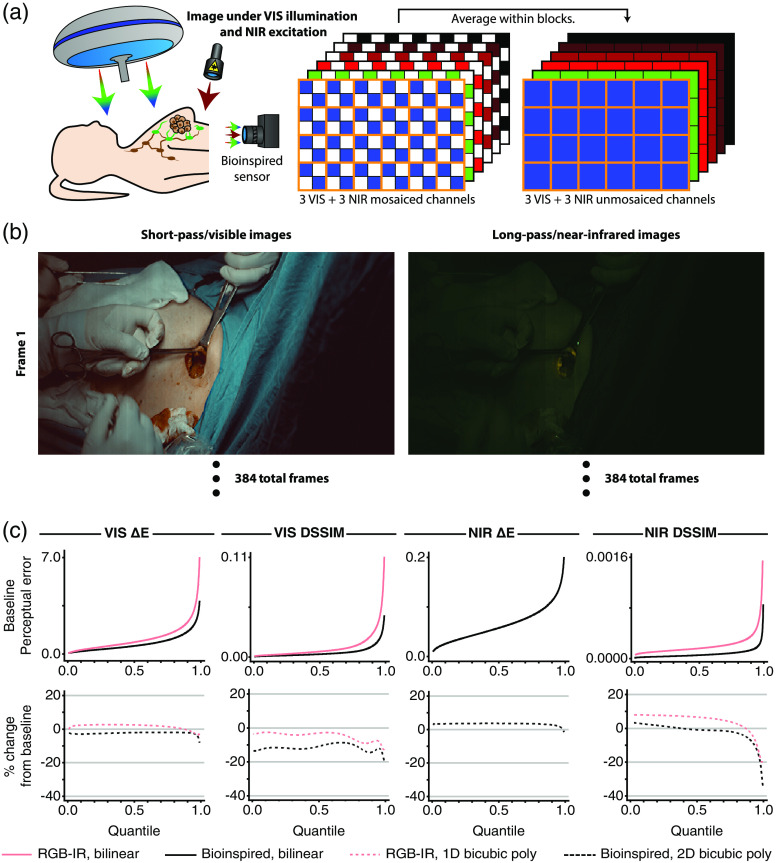
The experimental setup and unmosaiced images for the clinical dataset along with quantile functions for the perceptual metrics. (a) Seven women with breast cancer were administered indocyanine green and methylene blue so that any sentinel lymph nodes could be identified with near-infrared fluorescence. Raw images were collected using a stacked photodiode image sensor equipped with a bioinspired filter array, with illumination provided by both a VIS light source and an NIR source. Unmosaiced images were then constructed by taking spatial averages within each channel across 2×2  pixel blocks. (In this illustration, the light sources and the image sensor have been positioned for conceptual clarity; during the actual experiment, though, the visible light source was positioned above the patient, whereas the near-infrared light sources and the image sensor were all positioned to the side of the patient.) (b) One unmosaiced video was included in the dataset, showing the surgeon identifying and removing a sentinel lymph node before validating resection with a gamma probe. The video consisted of 384 frames and 640×368  pixels; the intensities in the visible frames and the near-infrared frames were scaled up by 1.5× and 3.0×, respectively, for display here. (c, top row) Quantile plots for visible color difference (VIS ΔE), visible dissimilarity index (VIS DSSIM), near-infrared color difference (NIR ΔE), and near-infrared dissimilarity index (NIR DSSIM) generated for an RGB-IR imaging system with bilinear demosaicing and a bioinspired imaging system with bilinear demosaicing. Each point indicates the proportion of pixels that have been assigned a value for a metric that is less than or equal to a given value for that metric. NIR ΔE could not be computed for the RGB-IR imaging system since only one of three total near-infrared channels could be captured by that camera. (c, bottom row) The percent change in the metric that was observed at each quantile after switching the RGB-IR imaging system from bilinear demosaicing to one-dimensional bicubic polynomial demosaicing and after switching the bioinspired imaging system from bilinear demosaicing to two-dimensional bicubic polynomial demosaicing.

A custom camera^28^ including a stacked photodiode image sensor with a bioinspired filter array was used to capture images; the camera was equipped with an emission filter (NF03-785E, Semrock). The patient was illuminated by a white light source (a surgical lamp with Cool Mirror Film 330, 3M) and a near-infrared source (BWF2-780-15-600-0.37-SMA, B&W Tek), and an image was captured—with short-pass observations at one-half of the pixels and long-pass observations at the other half of the pixels [Fig. 4(a)]. Spatial averages for each channel were then computed in nonoverlapping blocks according to Ref. 50 and a preprocessing routine was applied to the result, providing a reduced-resolution unmosaiced image with a short-pass observation and a long-pass observation at every pixel [Figs. 4(a), Supplementary Material].

This process yielded one unmosaiced video that was included in the dataset [Fig. 4(b)], showing a surgeon identifying and removing a sentinel lymph node and validating resection with a gamma probe through an incision in the patient. The video consisted of 384 frames and 640×368  pixels. Production of two mosaiced videos, computation of a set of demosaiced videos, and comparison with the unmosaiced video then followed those steps applied to the preclinical dataset.

### 6.2 Data Analysis

Tables 3 and 4 present the sum of absolute errors, mean absolute errors, structural dissimilarities, and color differences when examining the clinical dataset across image sensors and demosaicing routines. As with the previous dataset, it can be confirmed that the bioinspired sensor outperforms the RGB-IR sensor in every metric; that being said, there are some changes to the demosaicing method that should be optimally paired with each image sensor.

**Table 3:** SAE for clinical dataset. Statistics were pooled from a single video containing 384 frames. Unmosaiced channels were constructed by illuminating the subject with white light and near-infrared light, capturing several frames using a stacked photodiode image sensor with a bioinspired filter array, and downsampling by a factor of two along the rows and columns.

Metric	Bioinspired sensor					RGB-IR sensor				
	1D bilinear	1D bicubic poly	1D bicubic spline	2D bicubic poly	2D bicubic spline	1D bilinear	1D bicubic poly	1D bicubic spline	2D bicubic poly	2D bicubic spline
SAE (VIS R)	$1.53\times 10^{5}$	$1.50\times 10^{5}$	$1.51\times 10^{5}$	$1.48\times 10^{5}$	$1.58\times 10^{5}$	$2.63\times 10^{5}$	$2.63\times 10^{5}$	$2.73\times 10^{5}$	$2.88\times 10^{5}$	$2.73\times 10^{5}$
SAE (VIS G)	$1.47\times 10^{5}$	$1.35\times 10^{5}$	$1.34\times 10^{5}$	$1.29\times 10^{5}$	$1.35\times 10^{5}$	$2.69\times 10^{5}$	$2.51\times 10^{5}$	$2.55\times 10^{5}$	$3.16\times 10^{5}$	$2.55\times 10^{5}$
SAE (VIS B)	$2.00\times 10^{5}$	$1.89\times 10^{5}$	$1.89\times 10^{5}$	$1.86\times 10^{5}$	$1.96\times 10^{5}$	$3.51\times 10^{5}$	$3.39\times 10^{5}$	$3.46\times 10^{5}$	$3.97\times 10^{5}$	$3.46\times 10^{5}$
SAE (NIR 1)	$2.17\times 10^{4}$	$2.16\times 10^{4}$	$2.19\times 10^{4}$	$2.16\times 10^{4}$	$2.33\times 10^{4}$	$3.72\times 10^{4}$	$3.77\times 10^{4}$	$3.90\times 10^{4}$	$3.84\times 10^{4}$	$3.90\times 10^{4}$
SAE (NIR 2)	$1.59\times 10^{4}$	$1.56\times 10^{4}$	$1.57\times 10^{4}$	$1.53\times 10^{4}$	$1.65\times 10^{4}$	—	—	—	—	—
SAE (NIR 3)	$1.50\times 10^{4}$	$1.48\times 10^{4}$	$1.49\times 10^{4}$	$1.46\times 10^{4}$	$1.58\times 10^{4}$	—	—	—	—	—

**Table 4:** MAE, 99th percentile DSSIM, and 99th percentile ΔE for clinical dataset. The bioinspired sensor could be evaluated under the full set of metrics since it could capture three visible channels and three near-infrared channels, but the RGB-IR sensor could only be evaluated under a subset of metrics since it could capture just three visible channels and one near-infrared channel. A dash (“—”) indicates those cases where a statistic could not be computed since a camera did not capture all relevant channels.

Metric	Bioinspired sensor					RGB-IR sensor				
	1D bilinear	1D bicubic poly	1D bicubic spline	2D bicubic poly	2D bicubic spline	1D bilinear	1D bicubic poly	1D bicubic spline	2D bicubic poly	2D bicubic spline
MAE (VIS R)	$4.72\times 10^{-3}$	$4.61\times 10^{-3}$	$4.67\times 10^{-3}$	$4.57\times 10^{-3}$	$4.86\times 10^{-3}$	$5.41\times 10^{-3}$	$5.40\times 10^{-3}$	$5.62\times 10^{-3}$	$5.91\times 10^{-3}$	$5.62\times 10^{-3}$
MAE (VIS G)	$4.55\times 10^{-3}$	$4.17\times 10^{-3}$	$4.12\times 10^{-3}$	$3.98\times 10^{-3}$	$4.18\times 10^{-3}$	$5.53\times 10^{-3}$	$5.16\times 10^{-3}$	$5.23\times 10^{-3}$	$6.50\times 10^{-3}$	$5.23\times 10^{-3}$
MAE (VIS B)	$6.17\times 10^{-3}$	$5.84\times 10^{-3}$	$5.84\times 10^{-3}$	$5.72\times 10^{-3}$	$6.05\times 10^{-3}$	$7.23\times 10^{-3}$	$6.97\times 10^{-3}$	$7.13\times 10^{-3}$	$8.18\times 10^{-3}$	$7.13\times 10^{-3}$
MAE (NIR 1)	$6.69\times 10^{-4}$	$6.67\times 10^{-4}$	$6.76\times 10^{-4}$	$6.67\times 10^{-4}$	$7.19\times 10^{-4}$	$7.65\times 10^{-4}$	$7.75\times 10^{-4}$	$8.02\times 10^{-4}$	$7.90\times 10^{-4}$	$8.02\times 10^{-4}$
MAE (NIR 2)	$4.90\times 10^{-4}$	$4.80\times 10^{-4}$	$4.85\times 10^{-4}$	$4.71\times 10^{-4}$	$5.10\times 10^{-4}$	—	—	—	—	—
MAE (NIR 3)	$4.62\times 10^{-4}$	$4.55\times 10^{-4}$	$4.60\times 10^{-4}$	$4.51\times 10^{-4}$	$4.88\times 10^{-4}$	—	—	—	—	—
DSSIM (VIS)	0.0452	0.0384	0.0372	0.0366	0.0408	0.1099	0.0963	0.0967	0.1515	0.0967
ΔE (VIS)	3.7984	3.5790	3.5750	3.4945	3.7430	6.9556	6.7596	6.9934	8.0249	6.9934
DSSIM (NIR)	0.0005	0.0004	0.0004	0.0003	0.0004	0.0015	0.0012	0.0012	0.0020	0.0012
ΔE (NIR)	0.1992	0.1960	0.1975	0.1961	0.2129	—	—	—	—	—

For the bioinspired sensor, two-dimensional bicubic polynomial interpolation minimized the sum of absolute errors and the mean absolute error for both the visible spectrum and the near-infrared spectrum. Meanwhile, two-dimensional bicubic polynomial interpolation again took first when evaluating the color difference and structural dissimilarity via the previously proposed average rank method. For the RGB-IR sensor, one-dimensional bicubic polynomial interpolation and bilinear interpolation minimized the actual error for the visible spectrum and the near-infrared spectrum, respectively. Likewise, one-dimensional bicubic polynomial interpolation once more took first when evaluating the perceived error.

The top row of Fig. 4(c) presents seven quantile functions corresponding to the four perceptual error metrics (visible color difference, visible structural dissimilarity, near-infrared color difference, and near-infrared structural dissimilarity) evaluated for the two sensors and their associated bilinear demosaicing routines. The bottom row of Fig. 4(c) then presents seven curves describing the percent change in the quantile function when transitioning from the bilinear demosaicing routine for a given sensor to either a one-dimensional bicubic polynomial demosaicing routine for an RGB-IR senor or a two-dimensional bicubic polynomial demosaicing routine for a bioinspired sensor.

As with the preclinical dataset, the quantile function associated with the bioinspired sensor’s visible color difference lies above the quantile function for the RGB-IR sensor except at the ∼1% of pixels with a <0.062 color difference, and the quantile functions associated with the bioinspired sensor’s visible structural dissimilarity and near-infrared structural dissimilarity lie above the quantile function for the RGB-IR sensor for all quantiles. Consequently, the transition to a bioinspired sensor from an RGB-IR sensor offers a maximum improvement of 45% for the visible color difference, 59% for the visible structural dissimilarity index, and 75% for the near-infrared structural dissimilarity index, with any impairment remaining completely imperceptible.

Since bilinear interpolation does not necessarily optimize performance, though, it is relevant to consider how the error changes during a transition from this baseline to a more sophisticated demosaicing routine. For the bioinspired camera, a transition from bilinear interpolation to two-dimensional bicubic polynomial interpolation yielded improvements in the visible spectrum, with the visible color difference and the visible structural dissimilarity decreasing across all quantiles. However, the same transition yielded mixed results in the near-infrared spectrum, with the near-infrared color difference increasing for the bottom 98% of pixels and the near-infrared structural dissimilarity increasing for the bottom 38% of pixels. For the RGB-IR camera, a transition from bilinear interpolation to one-dimensional bicubic polynomial interpolation did not yield a decisive improvement in either region of the spectrum. While the visible structural dissimilarity decreased for all of the pixels, the visible color difference increased for 87% of the pixels; furthermore, the near-infrared structural dissimilarity also increased for 87% of the pixels. It is worth noting that the change in the perceived error during this transition between demosaicing routines was more negative for the bioinspired sensor than the RGB-IR sensor across all quantiles, indicating that such a transition is more likely to benefit the bioinspired sensor than the RGB-IR sensor. It is also worth noting that the change in the perceived errors trended negative for both sensors at the largest quantiles, indicating that pixels with low error might suffer from the transition but that pixels with high error were likely to benefit.

## 7 Conclusion

To maximize the patient’s outcome and the surgeon’s experience, the imaging systems used for near-infrared fluorescence image-guided surgery must capture multiple spectral channels spanning the visible and near-infrared and must present those spectral channels across a large field of view and with a small spatial resolution. However, division-of-focal-plane imaging systems, which are quick, compact, and free from coregistration error, are also characterized by a tradeoff between the channel count on one hand and the field of view and spatial resolution on the other.

Some choices made during the design of the imaging system can have an obvious effect on this tradeoff, permitting a larger number of channels to be observed across a smaller number of pixels, while other choices made during the implementation of the demosaicing routine may have a subtler effect, enabling the recovery of some features from sparse observations. Unfortunately, the combined impact of the imaging system and the demosaicing routine depends on the specifics of the scene, including the types of structures that are present and the location and orientation of those structures in the field of view.

To permit an exploration of the design space for imaging systems, this study presented combinations of two imaging systems and five demosaicing routines, each combination providing a fixed number of spectral channels, and applied those combinations across two datasets of different clinical relevance, each dataset with a fixed field of view. With the spectral channels and the field of view fixed in this way, the spatial resolution would vary for each combination of imaging system and demosaicing routine and could be observed in the error between the ground-truth (or unmosaiced) image of the scene and the estimated (or demosaiced) image of the scene.

In preclinical and clinical contexts, the performance of a bioinspired imaging system, which utilizes three layers of photodiodes and two types of spectral filters, exceeded that of RGB-IR imaging systems, which utilize one layer of photodiodes and four types of spectral filters, in both the spectral domain (channel count) and the spatial domain (field of view and spatial resolution). Not only did the bioinspired imaging system capture six channels when the RGB-IR imaging system captured only four, but the bioinspired imaging system also produced more accurate images of a scene than the RGB-IR imaging system across every single metric. Notably, this difference in accuracy was observed in both absolute measures of error and perceptual measures of error, indicating that a computer program that is sensitive to an absolute change in the image and a human observer that is sensitive to a perceived change in the image will both benefit from an improvement to the imaging system.

The performance of the bioinspired sensor and the RGB-IR sensor was optimized in many cases with a bicubic polynomial demosaicing routine, with the bioinspired sensor favoring a two-dimensional routine and the RGB-IR sensor favoring a one-dimensional routine; however, the results indicate that no demosaicing routine performs optimally in all contexts, with the bioinspired sensor being better served by a one-dimensional bicubic polynomial routine in some cases and the RGB-IR sensor being better served by a bilinear routine (Table S1 in the Supplementary Material). This suggests that performance may be optimized by selecting a different demosaicing routine for different channels, or even for different regions of interest—using, e.g., a more complex two-dimensional bicubic polynomial routine for channels or regions with high signal-to-noise ratios, saturated colors, or complex textures and a simpler bilinear routine elsewhere. Selection of the routine, or blending between routines, could be accomplished with a simple learned model.

From an engineering perspective, it is more difficult to transition from a traditional RGB-IR image sensor to a bioinspired image sensor than it is to migrate from a simple bilinear demosaicing routine to a bicubic polynomial demosaicing routine. However, the increase in effort is commensurate with the decrease in error, with changes to the image sensor yielding a greater reduction in the mean absolute error than changes to the demosaicing routine. Notably, the pixels that had experienced the largest reduction in error were the pixels that had already exhibited the largest error. A large error between the images indicates an abrupt change in the scene, which may, in turn, indicate an abrupt change in a tissue; as a result, the pixels with the largest errors are likely to be the pixels with the most relevance, making the large reduction in error invaluable to the clinical staff.

Of course, the RGB-IR image sensor benefits from a technological head start over the bioinspired image sensor that permits RGB-IR pixels to be constructed with smaller sizes and larger fill factors than bioinspired pixels. This gives the RGB-IR image sensor a built-in advantage over the bioinspired image sensor when it comes to spatial resolution or field of view, which was not accounted for in this study. Nonetheless, there is substantial room for improvement in the enabling technologies for the bioinspired image sensor that suggests ample space for engineers interested in closing this performance gap.

## Code, Data, and Materials Availability

All results associated with this study are present in the paper. The code used in the analyses is available in Ref. 52, while the data consumed in the analyses is available in Ref. 53. No materials were used under a material transfer agreement or other licenses.
