# Supplementary material for: Decoupling channel count from field of view and spatial resolution in single-sensor imaging systems for fluorescence image-guided surgery
Source: J Biomed Opt. 2022 Sep 26;27(9):096006. doi: 10.1117/1.JBO.27.9.096006 (PMC9511017; doi:10.1117/1.JBO.27.9.096006)
Supplement: Supplementary file 1 [file JBO_027_096006_SD001.pdf]

Title:

Supplementary Material for "Decoupling channel count from field of view and spatial resolution in single-sensor imaging systems for fluorescence image-guided surgery"

Authors:

Steven Blair, Missael Garcia, Zhongmin Zhu, Zuodong Liang, Benjamin Lew, Mebin George, Borislav Kondov, Sinisa Stojanoski, Magdalena Bogdanovska Todorovska, Daniela Miladinova, Goran Kondov, and Viktor Gruev

Contents:

Supplemental Materials and Methods

Pre-Processing of Images

Foreground Selection for Statistics

Table S1: Optimal combinations of sensors and demosaicing routines across different datasets and metrics.

Supplemental Materials and Methods

Pre-Processing of Images

Images underwent a limited series of pre-processing steps that were consistent with standard practices in the image processing community. Visible images underwent color correction in which pixels were transformed from a sensor-dependent RGB color space to a sensor-independent sRGB color space via a linear transformation from the RGB space to the CIEXYZ space and a nonlinear transformation from the CIEXYZ space to the sRGB space. Visible images and near-infrared images then underwent speckle correction in which pixels that appeared abnormally bright or abnormally dark when compared to neighboring pixels under a gaussian distribution were brought in line with those neighboring pixels.

Foreground Selection for Statistics

Statistics, including sums, means, and percentiles, incorporated a subset of pixels identified as the foreground while ignoring the subset of pixels identified as the background. A region of interest containing no content was selected by hand; the visible channels within this region of interest were then converted from a color response to a luminance response by transforming from the sRGB color space to the CIELAB color space before retaining the luminance component and discarding the chrominance components. An estimate of the sensor offset was determined by computing the spatiotemporal average over the region of interest; furthermore, an estimate of the sensor's temporal noise was determined by computing the spatial average of the temporal standard deviations over the region of interest, and an estimate of the sensor's spatial noise was determined by computing the spatial standard deviation of the temporal averages over the region-of-interest. A binary mask was formed from the image by activating those pixels that fell above the noise floor and deactivating those pixels that fell below, with the noise floor defined as the sum of the offset, six times the temporal noise, and six times the spatial noise. To exclude stuck-on pixels surrounded by otherwise dark pixels, the mask underwent morphological opening via two cycles of binary erosion and two cycles of binary dilation, and to include stuck-off pixels surrounded by otherwise bright pixels, the mask underwent region filling via the method in Ref. 51. To ensure that the boundary between the background and foreground was counted in the statistics, the mask also underwent morphological dilation via five cycles of binary dilation. Those pixels in the image that corresponded to activated pixels in the resulting mask were counted as foreground pixels that could be incorporated into the statistics, while those pixels that corresponded to deactivated pixels were counted as background pixels that had to be ignored in the statistics.

Table S1: Optimal combinations of sensors and demosaicing routines across different datasets and metrics. The optimum combination for the actual error was determined by assigning a rank to each demosaicing routine according to its performance on the sum of absolute errors or the mean absolute error, with the sum or the mean computed over either the pooled visible channels (VIS) or the pooled near-infrared channels (NIR). The optimum combination for the perceived error was determined by assigning an individual rank to each demosaicing routine according to its performance on each perceptual metric and computing an average rank for each demosaicing routine over all perceptual metrics (VIS+NIR). The bioinspired sensor outperformed the RGB-IR sensor in every metric, so the bioinspired sensor is preferred when any sensor can be used; however, recommendations for the demosaicing routine are provided for both the bioinspired sensor and the RGB-IR sensor in case there are any restrictions on the available sensor.

**Dataset**	**Metric**	**Optimal Sensor + Demosaicing Routine (Across Both Sensors)**	**Optimal Demosaicing Routine For Bioinspired Sensor**	**Optimal Demosaicing Routine For RGB-IR Sensor**
**Preclinical**	**Actual Error (VIS)**	Bioinspired + 2D bicubic polynomial	2D bicubic polynomial	1D bicubic polynomial
	**Actual Error (NIR)**	Bioinspired + 1D bicubic polynomial	1D bicubic polynomial	1D bicubic polynomial
	**Perceived Error (VIS+NIR)**	Bioinspired + 1D bicubic polynomial	1D bicubic polynomial	1D bicubic polynomial
**Clinical**	**Actual Error (VIS)**	Bioinspired + 2D bicubic polynomial	2D bicubic polynomial	1D bicubic polynomial
	**Actual Error (NIR)**	Bioinspired + 2D bicubic polynomial	2D bicubic polynomial	1D bilinear
	**Perceived Error (VIS+NIR)**	Bioinspired + 2D bicubic polynomial	2D bicubic polynomial	1D bicubic polynomial
